# Supplementary figures and images for: Predicting Antimicrobial and Other Cysteine-Rich Peptides in 1267 Plant Transcriptomes
Source: Antibiotics (Basel). 2020 Feb 4;9(2):60. doi: 10.3390/antibiotics9020060 (PMC7168108; doi:10.3390/antibiotics9020060)

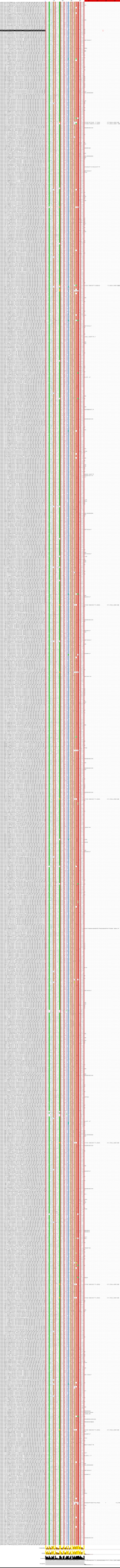

Supplement: Supplementary file 1 [file antibiotics-09-00060-s001.zip › figS1.png]
